# Filaricidal activity of *Daniellia oliveri* and *Psorospermum febrifugum* extracts

**DOI:** 10.1186/s13071-021-04759-6

**Published:** 2021-06-07

**Authors:** Melanie Abongwa, Moses Samje, Godfred A. Ayimele, Smith B. Babiaka, Christina Bulman, Judy Sakanari, Nick J. Koszewski, Saurabh Verma, Jesse Goff, Fidelis Cho-Ngwa, Richard J. Martin, Alan P. Robertson

**Affiliations:** 1 Department of Biomedical Sciences, College of Veterinary Medicine, Iowa State University, Ames, IA USA; 2 ANDI Centre of Excellence for Onchocerciasis Drug Research, Faculty of Science, University of Buea, South West Region, Buea, Cameroon; 3 Department of Biomedical Sciences, Faculty of Health Sciences, University of Bamenda, Bamenda, Cameroon; 4 Department of Chemistry, Faculty of Science, University of Buea, South West Region, Buea, Cameroon; 5 Center for Discovery and Innovation in Parasitic Diseases, University of California San Francisco, San Francisco, CA USA

**Keywords:** Onchocerciasis, Lymphatic filariasis, *Daniellia oliveri*, *Psorospermum febrifugum*, Cytotoxicity

## Abstract

**Background:**

Drugs currently used for controlling onchocerciasis and lymphatic filariasis (LF) are mainly microfilaricidal, with minimal or no effect on the adult worms. For efficient management of these diseases, it is necessary to search for new drugs with macrofilaricidal activities that can be used singly or in combination with existing ones. *Daniellia oliveri* and *Psorospermum febrifugum* are two plants commonly used in the local management of these infections in Bambui, a township in the North West Region of Cameroon, but there is currently no documented scientific evidence to support their claimed anthelmintic efficacy and safety. The aim of this study was to provide evidence in support of the search for means to eliminate these diseases by screening extracts and chromatographic fractions isolated from these plants for efficacy against the parasitic roundworms* Onchocerca ochengi* and *Brugia pahangi.*

**Methods:**

The viability of *O. ochengi* adult worms was assessed using the MTT/formazan assay. Fully confluent monkey kidney epithelial cells (LLC-MK2) served as the feeder layer for the *O. ochengi* microfilariae (mfs) assays. Viability of the mfs was assessed by microscopic examination for mean motility scoring (relative to the negative control) every 24 h post addition of an extract. The Worminator system was used to test the effects of the extracts on adult *B. pahangi* motility, and mean motility units were determined for each worm. Cytotoxicity of the active extracts on N27 cells was assessed using the MTS assay.

**Results:**

Extracts from *D. oliveri* and *P. febrifugum* were effective against the adult roundworms *O. ochengi* and *B. pahangi*. Interestingly, extracts showing macrofilaricidal activities against *O. ochengi* also showed activity against *O. ochengi* mfs. The hexane stem bark extract of *D. oliveri* (DO_BHEX_) was more selective for adult *O. ochengi* than for mfs, with a half maximal and 100% inhibitory concentration (IC_50_ and IC_100_, respectively) against adult *O. ochengi* of 13.9 and 31.3 μg/ml, respectively. The* in vitro* cytotoxicity of all active extracts on N27 cells showed selective toxicity for parasites (selectivity index > 1). Bioassay-guided fractionation of the extracts yielded fractions with activity against adult *B. pahangi*, thus confirming the presence of bioactive principles in the plant extracts.

**Conclusions:**

Our study supports the use of *D. oliveri* and *P. febrifugum* in the traditional treatment of onchocerciasis and LF. The further purification of active extracts from these plants could yield lead compounds for filarial drug discovery and development.

**Graphic abstract:**

**Figure:**
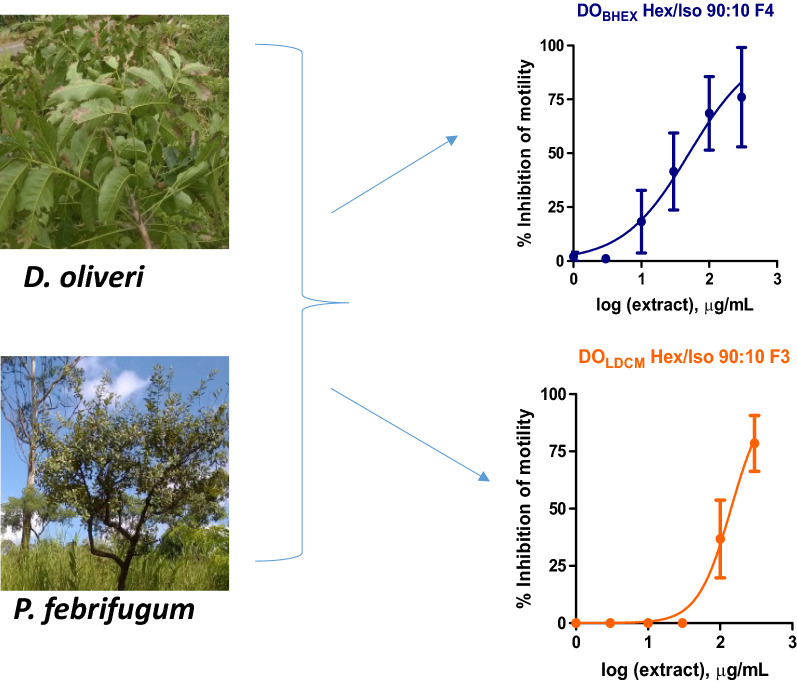

**Supplementary Information:**

The online version contains supplementary material available at 10.1186/s13071-021-04759-6.

## Background

Onchocerciasis, also known as subcutaneous filariasis or river blindness, and lymphatic filariasis (LF), commonly known as elephantiasis, are currently classified by the World Health Organization as “neglected tropical diseases” (NTDs) [1]. Onchocerciasis is caused by the filarial nematode parasite *Onchocerca volvulus* and transmitted by the bite of the blackfly, *Simulium damnosum* [2]. Approximately 37 million people globally suffer from onchocerciasis, the majority of whom are in Africa where > 100 million people are at high risk of infection [3]. Onchocerciasis is the world’s second leading infectious cause of blindness, after trachoma. In Africa, hyperendemic villages can have extremely high infection rates, while > 10% of an entire village may be blind [4]. Additionally, onchocerciasis presents a spectrum of other debilitating pathologies or conditions which include grave and unbearable itching, disfiguring dermatitis and depigmentation [5].

LF is caused by the filarial nematode parasites *Brugia malayi*, *B. timori* and *Wuchereria bancrofti*; they are transmitted by a variety of mosquitoes (*Anopheles *spp., *Culex *spp., *Aedes *spp., *Mansonia *spp.) depending on the geographical area [6]. The disease manifestations include painful and profound disfigurement of the skin (particularly of the lower portion of the body), lymphedema, elephantiasis and scrotal swelling which may eventually lead to permanent disability [7]. LF is the second leading cause of chronic disability worldwide, after mental illness. An estimated > 1.2 billion people are threatened by LF, with > 120 million people infected and approximately 40 million people disfigured and incapacitated by the disease [8, 9]. Hence, individuals infected with either onchocerciasis and/or LF not only suffer physical disability, but also suffer mental, social and economic losses, thus resulting in depression, stigma and poverty [10–12].

Current control of onchocerciasis and LF relies on mass drug administration of ivermectin, diethycarbamazine (DEC) and albendazole [13–15]. However, these drugs are only effective against the juvenile worms (microfilariae [mfs]) or larval stages of the parasites, with limited activity against the adult worms (macrofilariae). This leaves the adult worms to continue to produce mfs and exacerbate the disease symptoms. Effective control of onchocerciasis and LF is therefore hindered by the lack of adulticides (macrofilaricides). The use of these drugs is also limited by factors, such as poor compliance and poor safety. Despite ≥ 15 years of mass treatment with ivermectin, transmission of onchocerciasis in some parts of Africa, such as in the North and West Regions of Cameroon, has still not been interrupted [16, 17]. Also, the emergence of ivermectin resistance in parasites of veterinary importance has generated the concern that resistance will eventually extend to *O. volvulus* [18, 19]. Current treatments can also cause life-threatening adverse reactions in patients with onchocerciasis and LF who are co-infected with *Loa loa*, a filarid nematode that causes* L. loa* filariasis. Patients with onchocerciasis and LF co-infected with *L. loa* may have very high levels of *L. loa* mfs. Treatment of these patients with currently available drugs can result in the sudden and massive death of *L. loa* mfs and cause serious adverse reactions, including encephalopathy, kidney failure and even death [20–23]. DEC is further contraindicated in patients with onchocerciasis due to the likelihood of severe exacerbation of reactions involving the skin and eye [24]. Therefore, there is a pressing need for new and safer filaricidal compounds to promote control and eradication of onchocerciasis and LF.

Historically, plants have been recognized as important sources of therapeutic compounds, especially in folk medicine [25–27]. About 70–80% of the population in developing countries rely on traditional medicine for their health needs [28]. The use of traditional medicine has evolved over the years and is no longer limited to developing countries; for example, in recent times, interest in natural therapy in developed countries such as the USA has flourished, including the use of herbal, dietary or nutritional supplements [29, 30]. There has also been a resurgence in exploring plant natural products for the development of antiparasitic drugs. Several studies on the antifilarial activity of plant extracts and chromatographic fractions of medicinal plants have been reported [31–33]. The results of these studies suggest that plant natural products may serve as sources of lead compounds for the development of new and safe macrofilaricides, while the development of new microfilaricides is also desirable.

*Daniellia oliveri* (DO) and *Psorospermum febrifugum* (PF) are among the plants commonly used for the traditional treatment of onchocerciasis and LF in Bambui, a township in the highlands of the North West Region of Cameroon, where the vegetation is predominantly of the forest savannah type. This village is located near many streams running down stony hill slopes, thus providing a good breeding site for arthropod vectors and transmission of infections, including onchocerciasis and LF. *Daniellia oliveri*, locally known in Bambui as ‘kahi’, is traditionally used for the treatment of all worm infections, including onchocerciasis and LF, and also for treating stomach upset. It is prepared by boiling the stem bark and is administered as a decoction of the stem bark, drinking one cup of the decoction twice daily. *Psorospermum febrifugum* is locally known in Bambui as ‘sawayki’ and is traditionally used to treat onchocerciasis and rashes of any sort. Additionally, the decoction can be used for bathing with and the oily supernatant applied as body lotion.

Despite the claimed efficacy of *D. oliveri* and *P. febrifugum* in the traditional treatment of onchocerciasis and LF, no scientific evidence for this claim has been documented. Therefore, the overall aim of this study was to screen extracts and chromatographic fractions of *D. oliveri* and *P. febrifugum* for activity against *Onchorcerca *sp. and *Brugia *sp. in an attempt to contribute to the search for suitable macrofilaricides to support the control or elimination of onchocerciasis and LF. Although the treatment of onchocerciasis and LF is greatly hampered by the lack of macrofilaricides, new and safer microfilaricides which will replace or complement currently available drugs are also needed. Hence, we also screened plant extracts for microfilaricidal activity. For this study, we used *O. ochengi* for screening for anti-*Onchocerca* activity because it is the best-known laboratory model for the human equivalent, *O. volvulus*. *Brugia pahangi* was used for screening for anti-*Brugia* activity because it is a suitable laboratory model organism for *B. malayi* [34].

## Methods

### Collection and identification of plants

Plants were collected from Bambui in Tubah Sub-Division, North West Region, Cameroon based on ethnopharmacological information. Voucher specimens of all plants harvested were prepared and taken to the National Herbarium in Yaounde and to the Limbe Botanic Garden in Limbe, Cameroon, for expert identification. The classification of the plants used in this study and other relevant information are listed in Additional file 1: Table S1.

### Preparation of crude extracts

The plant parts collected were air-dried in Bambui before transport to the University of Buea (Buea, South West Region, Cameroon) where they were further dried at 40 °C for 48 h and then ground to fine powder using an electric blender (leaves) or a grinding mill (stem bark). The resultant powders were weighed and sequentially macerated with technical grade hexane (HEX), dichloromethane (DCM) and methanol (MeOH), such that the plant material was always fully submerged in solvent. For each solvent, the slurry was allowed to stand in a sealed container at room temperature for 48 h and then filtered through No. 1 Whatman filter paper (Sigma-Aldrich, St. Louis, MO, USA). Extracts were concentrated under reduced pressure on a rotary evaporator (Buchi Rotavapor R-200; Buchi AG, Flawil, Switzerland). Concentrated extracts were transferred into vials and allowed to stand open at room temperature until all residual solvent had evaporated. The resultant crude extracts were then weighed, and their respective percentage (%) yield determined (Additional file 1: Table S2) using the formula:

$$\% \, yield \, = \, \left[ {\left( {\text{Weight of crude extract}} \right) \div \left( {\text{Dry weight of plant part}} \right)} \right] \, \times \, 100.$$

Vials were then sealed and stored at – 20 °C or – 80 °C until needed for subsequent screening.

### Bioassay-guided fractionation of extracts

Crude extracts were tested in all three biological assays (Section Biological assays) to determine activity prior to fractionation.

#### Sep-Pak solid-phase extraction

Bioassay-guided fractionation was performed on the three most active extracts using solid-phase extraction (SPE) with Sep-Pak Silica (normal phase) and C18 (reversed phase) Plus cartridges (Waters Corp., Milford, MA, USA). Sep-Pak Silica Plus cartridges were used for non-polar (HEX) extracts, using stepwise gradient elution in HEX:isopropanol mixtures (100:0, 100:0, 90:10, 50:50, 10:90), and a final elution in 100% chloroform (CHCl_3_). For polar (MeOH) extracts, we used Sep-Pak C18 Plus cartridges, with stepwise gradient elution in MeOH:H_2_O mixtures (20:80, 20:80, 40:80, 60:80, 80:20, 100:0). For DCM extracts whose polarity is midway between HEX and MeOH, two separate extractions were performed with each of the Sep-Pak cartridges. For both cartridges, the ratio of extracts to solvent system was always 2:1, which was achieved by re-suspending 10 mg of extract in 5 ml of the appropriate solvent system. The resulting Sep-Pak fractions generated were separately concentrated using in-house air at a maximum temperature of 40 °C.

#### Semi-preparative high-performance liquid chromatography

Active Sep-Pak fractions were selected for further purification using a high-performance liquid chromatography (HPLC) system (HP 1050 HPLC System; Hewlett-Packard Co., Palo Alto, CA, USA) coupled with a Hewlett Packard/Agilent 1050 Diode Array Detector (DAD)/Multiple Wavelength UV Detector (MWD) and silica (straight phase) semi-preparative column (SUPELCOSIL LC-SI SEMI-PREP, 25 cm × 10 mm, 5 µm; Hewlett-Packard Co., catalog no. 58365). Elution was performed with a 100 to 0% gradient of 99.6% HEX in 0.4% isopropanol (solvent A) and a 0–100% gradient of 50% HEX in 50% isopropanol (solvent B) for 30 min, with an additional collection of the eluent for 5 min, at a flow rate of 4 ml/min to produce seven sub-fractions (F1–F7), collected every 5 min. Fractions were detected with DAD/MWD at 220, 264 and 320 nm.

### Biological assays

#### Adult* B. pahangi* motility assay

The *B. pahangi* assays were carried out using the Worminator worm motility tracking system as previously described [35]. Adult female *B. pahangi* were shipped from the University of Missouri-Columbia (Columbia, MO, USA) or from the NIAID/NIH Filariasis Research Reagent Resource Center (FR3; Athens, GA, USA) in media (RPMI-1640 with 25 mM HEPES, 2.0 g/l NaHCO_3_, 5% heat-inactivated fetal bovine serum [FBS] and 1× antibiotic/antimycotic solution). The worms were observed under an inverted microscope to ascertain their viability. Damaged or sluggish worms were discarded, and only highly motile worms were retained for the assays. For the assays, 30 mg/ml stocks of extracts or chromatographic fractions were prepared in DMSO and tested in quadruplicate at an initial working concentration of 300 µg/ml (1% DMSO). The negative control wells contained 1% DMSO and positive control wells contained 10 µM auranofin. The worms were cultured in a 5% CO_2_ incubator at 37 °C for 72 h post addition of the chosen drug.

To test the effects of the extracts or chromatographic fractions on worm motility, the Worminator system was used to record videos of each worm plate for approximately 60 s (at 0, 4, 24, 48 and 72 h post addition of drug). Mean motility units (MMUs) (number of pixels displaced per second) were determined for each worm. Percentage inhibition of motility was calculated as:

$${1}00 - {1}00\left[ {\left( {\text{MMUs of test worm}} \right) \div \left( {\text{Average MMUs of the controls}} \right)} \right].$$

Extracts which showed ≥ 95% inhibition of motility or chromatographic fractions which showed ≥ 75% inhibition of motility were considered to be active and further screened under the same conditions at six serially diluted concentrations (300, 100, 30, 10, 3 and 1 µg/ml) to determine the respective half maximal inhibitory concentration (IC_50_).

#### *Onchocerca ochengi* microfilariae assay

Extraction of *O. ochengi* microfilariae (mfs) followed the method of Cho-Ngwa et al. [31] with minor modifications. Fresh pieces of umbilical cattle skin containing palpable nodules were obtained from the slaughterhouse in Douala, Littoral Region, Cameroon, and immediately transported to the laboratory. The skin was thoroughly washed twice with soap and distilled water, drained and transferred to a sterile laminar flow hood where the skin was entirely covered with 70% ethanol that was subsequently allowed to evaporate completely. Autoclaved thumb tacks were used to attach the skin tightly to a clean wooden board with the outer surface of the skin facing upwards. The outer surface was shaved with a sterile blade and then rinsed twice with distilled water. Excess moisture from the skin was removed by dabbing with a clean dry cloth. The entire skin was covered with 70% ethanol and allowed to evaporate in a laminar flow hood. Skin snips approximately 0.5–1 mm apart were obtained from different locations of the skin. The skin snips were placed in 20 ml of complete culture medium (CCM: RPMI-1640 supplemented with 25 mM HEPES, 2.0 g/l sodium bicarbonate, 20 mM l-glutamine, 10% newborn calf serum, 200 units/ml penicillin, 200 μg/ml streptomycin and 2.5 μg/ml amphotericin B, pH 7.4) and incubated at room temperature for 2 h to allow for the emergence of mfs. Highly motile emerged mfs were selected and concentrated centrifugation under mild conditions (400  *g*, 10 min). The supernatant was decanted and the pellet resuspended in fresh CCM. Mfs were diluted to approximately 100–150/ml. Thereafter, 100-µl aliquots of CCM containing mfs were distributed into 96-well culture plates to obtain an average of 10–15 mfs per well.

Fully confluent monkey kidney epithelial cells (LLC-MK2) obtained from American Type Culture Collection (ATCC, Manassas, VA, USA) served as the feeder layer for the mfs. The cultures were maintained at 37 °C in a 5% CO_2_ incubator (HERACell 150 System; Haraeus, Hanau, Germany). Extract stock solutions in DMSO (25 mg/ml) were prepared and tested in duplicate at eight serially diluted concentrations, ranging from 250 to 1.95 µg/ml in a final CCM volume of 200 µl. A 1% DMSO solution was used as the negative control while 10 µg/ml ivermectin served as the positive control. At 10 µg/ml, ivermectin produced 100% inhibition of mfs motility at 120 h post addition. Mfs viability was assessed by microscopic examination for mean motility scoring (relative to the positive control) every 24 h post addition of drug for 120 h. The scale used for motility scoring ranged from 0 to 100% (100% for complete inhibition, 75% for only head or tail movement, 50% for entire worm moving sluggishly, 25% for little change in motility, 0% for no movement). An extract was considered active if it produced 100% inhibition of mfs motility at the highest concentration tested (250 µg/ml). The serial dilutions allowed for the determination of IC_50_ values.

#### *Onchocerca ochengi* macrofilariae assay

Viable *O. ochengi* adult worm (macrofilariae) masses were extracted from the cattle skin as previously described [31]. Briefly, infected cattle skin was obtained, cleaned and sterilized as described in preceding section. Adult worm masses, which are generally pale orange–yellow in appearance, were extracted from the nodules by careful dissection with a sterile blade. Extracted worms were transferred to sterile 24-well culture plates containing 1 ml/well CCM, and incubated at 37 °C in a 5% CO_2_ incubator (HERACell 150 system; Haraeus) overnight. The viability of extracted adult worms was ascertained by microscopic examination using an inverted microscope (Euromex Microscopen B.V., Arnhem, The Netherlands). Dead and damaged worms were discarded, and only viable worms were retained for the assays.

Stock solutions of each extract were prepared in DMSO (25 mg/ml). Extracts were initially tested in triplicate at a working concentration of 500 µg/ml in order to eliminate inactive extracts; 1% DMSO and 10 µM auranofin were used as negative and positive controls, respectively. Macrofilariae cultures were incubated at 37 °C in a 5% CO_2_ incubator for 120 h, without any change in CCM. Cultures were terminated at the end of the 120-h incubation period and adult worm viability was assessed using the MTT/formazan assay [36]. Each nodular worm mass was placed in each well of a 48-well plate containing 500 µl of 0.5 mg/ml MTT [3-(4,5-dimethylthiazol-2-yl)-2,5-diphenyltetrazolium bromide] in incomplete culture medium (CCM without serum). The plates were incubated in the dark at 37 °C for 30 min, after which individual worms were blotted on tissue paper and transferred to a clean white sheet of paper. Percentage inhibition of formazan formation, as indicated by development of purple coloration, relative to the negative control was then visually estimated. Mean percentage inhibition of formazan formation, as indicated by the deep purple coloration, relative to the negative control worm masses correlates with worm death. Extracts that showed 100% inhibition of formazan formation were considered to be active and further tested at six different serial dilutions from 500 µg/ml to 15.6 µg/ml to determine IC_50_ values.

### Mammalian cytotoxicity using the MTS assay

Cytotoxicity was assessed to evaluate the quality of the active extracts which were toxic to N27 cells, an immortalized rat mesencephalic neuronal cell line. The IC_50_ values obtained for the cells were then compared to the IC_50_ values obtained for the parasites (IC_50_ N27 cell/IC_50_ parasite) to determine how selective the active extracts are for the parasites over the cells (selectivity index [SI]). In this study, cytotoxicity was assessed using the MTS [3-(4,5-dimethylthiazol-2-yl)-5-(3-carboxymethoxyphenyl)-2-(4-sulfophenyl)-2H-tetrazolium, inner salt] assay [37]. N27 cells were seeded into 96-well culture plates at 5000 cells/100 µl of RPMI growth media (RPMI-1640 medium containing 10% FBS, 2 mM l-glutamine, 50 units penicillin, and 50 µg/ml streptomycin). Treatment with active extracts followed once the cells had achieved 40–50% confluence. All treatments were performed in sextuplicate; 30 µM H_2_O_2_ served as the positive control, and the negative control wells contained cells and growth media containing 1% DMSO. Plates were incubated at 37 °C in a 5% CO_2_ incubator (Forma Scientific Series; Thermo Fisher Scientific, Waltham, MA, USA) for 24 h. A 10-µl aliquot of MTS reagent (CellTiter 96® Aqueous One Solution Reagent; Promega, Madison, WI, USA, catalog number G3580) was then added to each well and mixed. The plates were covered with aluminum foil and incubated at 37 °C in a 5% CO_2_ incubator (Forma Scientific Series) for 90 min, after which they were immediately read at 490 nm and 670 nm using a SpectraMax M2® reader (Molecular Devices, San Jose, CA, USA). Cell viability was assessed as Abs_490_Abs_670nm_ relative to the untreated controls. Extracts with SI values > 1 were considered to be potentially useful.

### Phytochemical screening

Concentrated crude extract residues were used to detect the presence of secondary plant metabolites, such as alkaloids, steroids, flavonoids, saponins, tannins, coumarins and cardiac glycosides, using standard methods with minor modifications [38].

#### Test for saponins (frothing test)

Briefly, saponins were tested by dissolving 0.5 g of the crude extract in a test tube containing 3 ml of hot distilled water, followed by vigorous shaking for 1 min. The observation of persistent foaming indicated the presence of saponins.

#### Test for flavonoids (cyanidine test)

Crude extract (0.5 g) was dissolved in approximately 3 ml MeOH, followed by the addition of 2 ml of concentrated hydrochloric acid. A spatula full of magnesium turnings was then added to the mixture. A brick red coloration with effervescence indicated the presence of flavonoids.

#### Test for steroids (Lieberman-Burchard test)

Crude extract (0.5 g) was dissolved in 5 ml DCM to give a dilute solute solution and then 0.5 ml of acetic anhydride was added, followed by three drops of concentrated sulphuric acid. Observation of a blue-green coloration indicated the presence of steroids.

#### Test for tannins (ferric chloride test)

Crude extract (0.5 g) was dissolved in a boiling tube containing 20 ml distilled water and then boiled for 1 h, following which three to five drops of ferric chloride were added and the solution allowed to stand for proper color development. A blue-black coloration indicated a positive test for tannins.

#### Test for alkaloids (Dragendorf’s test)

The sample was dissolved in about 3 ml of DCM and then spotted on a thin layer chromatographic plate which was developed in 20% HEX in ethyl acetate. The presence of alkaloids in the developed chromatogram was detected by spraying with freshly prepared Dragendorf’s reagent in a fume chamber. A positive reaction on the chromatogram was indicated by an orange or darker colored spot against a yellow background, confirming the presence of alkaloids.

#### Test for coumarins

A 1-ml aliquot of ammonium hydroxide was added to 2 ml of the crude extract. One drop was spotted on filter paper and observed under a UV lamp for the presence of blue or green fluorescence which would indicate the presence of coumarins.

#### Test for cardiac glycosides

Approximately 1 ml of aqueous extract of the plants was added to 2 ml of glacial acetic acid plus one drop of ferric chloride. The solution was transferred to a test tube containing 1 ml of concentrated sulfuric acid. The appearance of violet and brownish rings below the interface, followed by the formation of a greenish ring in the acetic acid layer confirmed the presence of cardiac glycosides.

### Data analysis

Results obtained for biological activity and cytotoxicity assays were analyzed using GraphPad Prism 5.0 or 6.0 software (GraphPad Software, San Diego, CA, USA), and expressed as mean ± standard error of the mean. Statistical analysis for the cytotoxicity assay was done using unpaired Student *t*-tests. A *P*-value < 0.05 was considered to indicate significance.

## Results

### Activity of crude extracts

#### Activity of crude extracts against adult* B. pahangi*

**Fig. 1:**
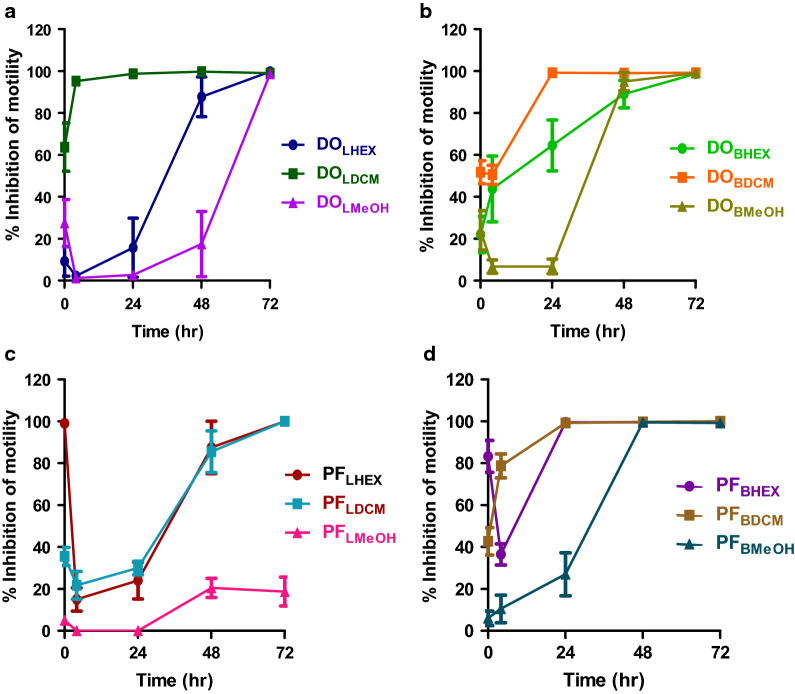
Time-dependent inhibition of adult *Brugia pahangi* motility following incubation with *Daniellia oliveri* (*DO*) and *Psorospermum febrifugum* (*PF*) leaf (*L*) and bark (*B*) extracts (*HEX* hexane,* DCM* dichloromethane,* MeOH* methanol). Results are plotted as the mean ± standard error of the mean (SEM), *n* = 4. All 12 extracts tested, with the exception of PF_LMeOH_, caused a 100% inhibition of motility after 72 h of incubation. DO_LDCM_ and DO_BDCM_ caused close to 100% inhibition of motility after 4 and 24 h of incubation

**Fig. 2:**
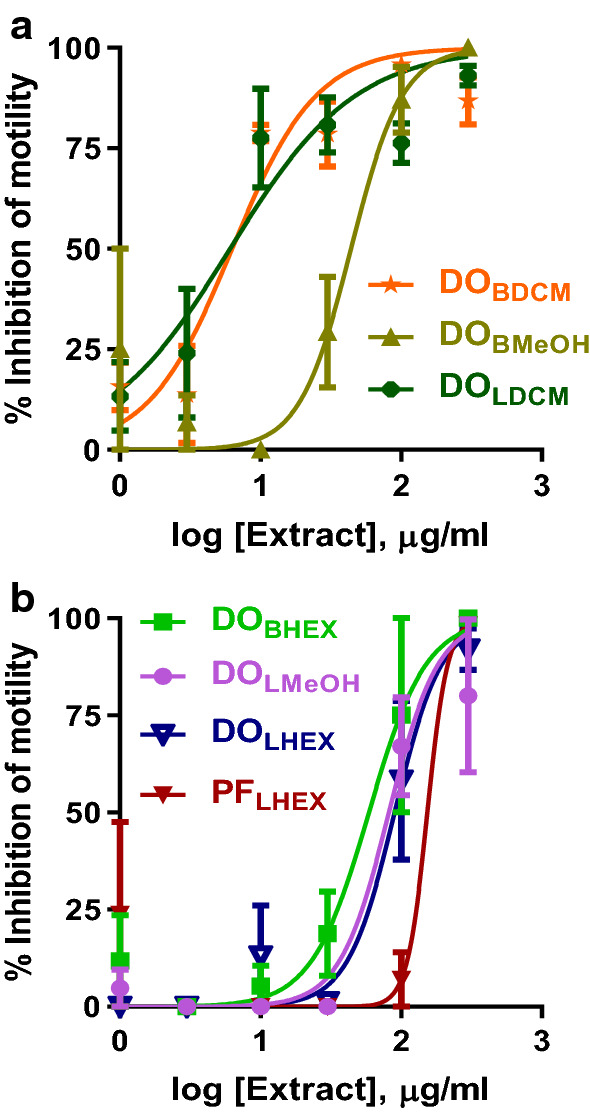
Concentration-dependent inhibition of adult *B. pahangi* motility after 72 h of incubation with *D. oliveri* and *P. febrifugum* extracts. Results are plotted as the mean ± SEM, *n* = 4. DO_LDCM_ (IC_50_ 6.1 ± 1.3 µg/ml) ≈ DO_BDCM_ (IC_50_ 6.3 ± 1.2 µg/ml) > > > DO_BMeOH_ (IC_50_ 44.0 ± 1.3 µg/ml) > DO_BHEX_ (IC_50_ 59.0 ± 1.2 µg/ml) > DO_LMeOH_ (IC_50_ 81.9 ± 1.2 µg/ml) ≈ DO_LHEX_ (IC_50_ 89.8 ± 1.3 µg/ml) > PF_LHEX_ (IC_50_ 153.7 ± 1.3 µg/ml).* IC*_*50*_ Half maximal inhibitory concentration

Of the 12 extracts initially screened for activity against adult *B. pahangi* at 300 µg/ml, 11 were active, while only extract PF_LMeOH_ (MeOH extract *P. febrifugum* [PF] leaves [L]) was inactive (Fig. 1a–d). The effects were also time dependent, with extracts DO_LDCM_ (DCM extract of *D. oliveri* [DO] leaves [L]) and DO_BDCM_ (DCM extract of *D. oliveri* [DO] bark [B]) causing close to 100% inhibition of motility after 4 and 24 h of incubation. Of these 11 active extracts, seven (DO_LHEX_, DO_LDCM_, DO_LMeOH_, DO_BHEX_, DO_BDCM_, DO_BMeOH_ and PF_LHEX_) were further screened for IC_50_ determination (Fig. 2). The rank order of the potency of these extracts on adult *B. pahangi* was: DO_LDCM_ (IC_50_ 6.1 ± 1.3 µg/ml, *n* = 4) ≈ DO_BDCM_ (IC_50_ 6.3 ± 1.2 µg/ml, *n* = 4) > > > DO_BMeOH_ (IC_50_ 44.0 ± 1.3 µg/ml, *n* = 4) > DO_BHEX_ (IC_50_ 59.0 ± 1.2 µg/ml, *n* = 4) > DO_LMeOH_ (IC_50_ 81.9 ± 1.2 µg/ml, *n* = 4) ≈ DO_LHEX_ (IC_50_ 89.8 ± 1.3 µg/ml, *n* = 4) > PF_LHEX_ (IC_50_ 153.7 ± 1.3 µg/ml, *n* = 4) (Table 1). IC_50_ values for the other four active extracts (PF_LDCM_, PF_BHEX_, PF_BDCM_ and PF_BMeOH_) are yet to be determined.

**Table 1:** Half maximal inhibitory concentrations of *Daniellia oliveri* and *Psorospermum febrifugum* plant extracts for *Onchocerca ochengi* and *Brugia pahangi*

Extracts	*O. ochengi*		*B. pahangi*
	IC_50_ microfilariae (µg/ml)^a^	IC_50_ adult worm (µg/ml)^a^	IC_50_ adult worm (µg/ml)^a^
DO_LHEX_	Inactive	Inactive	89.8 ± 1.3
DO_LDCM_^b^	16.2 ± 0.0	43.3 ± 9.2	6.1 ± 1.3
DO_LMeOH_	Inactive	Inactive	81.9 ± 1.2
DO_BHEX_^b^	185.2 ± 0.0	13.9 ± 1.7	59.0 ± 1.2
DO_BDCM_^b^	9.7 ± 0.0	22.5 ± 0.0	6.3 ± 1.2
DO_BMeOH_	Inactive	Inactive	44.0 ± 1.3
PF_LHEX_	Inactive	Inactive	153.7 ± 1.5
PF_LDCM_	132.4 ± 0.0	Inactive	Yet to be determined
PF_LMeOH_	8.8 ± 0.0	Inactive	Inactive
PF_BHEX_	4 ± 0.0	Inactive	Yet to be determined
PF_BDCM_	7.2 ± 0.0	Inactive	Yet to be determined
PF_BMeOH_	6.6 ± 0.0	Inactive	Yet to be determined

*DO* *Daniellia oliveri*,* PF* *Psorospermum febrifugum*; *HEX* hexane,* DCM* dichloromethane,* MeOH* methanol

^a^Half maximal inhibitory concentration (IC_50_) is given as the mean ± standard error of the mean

^b^Extracts DO_BHEX_, DO_LDCM_ and DO_BDCM_ were the only plant extracts of the 12 active extracts identified which were active in all three biological assays used in this study (*O. ochengi* microfilariae, adult *O. ocheng*i or adult *B. pahang*)

#### Activity of crude extracts against* O. ochengi* mfs

For the *O. ochengi* mf assay, eight of the 12 extracts screened showed a concentration-dependent inhibition of mf motility, namely the HEX extract of *D. oliveri* stem bark (DO_BHEX_), the DCM extracts of *D. oliveri* leaves (DO_LDCM_), the DCM extracts of *D. oliveri* bark (DO_BDCM_), the DCM leaf extracts of *P. febrifugum* (PF_LDCM_), the MeOH leaf extract of *P. febrifugum* (PF_LMeOH_), the HEX stem bark extract of *P. febrifugum* (PF_BHEX_), the DCM stem bark extract of *P. febrifugum* (PF_BDCM_) and the MeOH stem bark extract of *P. febrifugum* (PF_BMeOH_). The effects of these extracts were also time dependent. The rank order potency for *O. ochengi* microfilaricidal effects was: PF_BHEX_ (IC_50_ 4 ± 0.0 µg/ml, *n*  = 2) > PF_BMeOH_ (IC_50_ 6.6 ± 0.0 µg/ml, *n*  = 2) > PF_BDCM_ (IC_50_ 7.2 ± 0.0 µg/ml, *n* = 2) > PF_LMeOH_ (IC_50_ 8.8 ± 0.0 µg/ml, *n* = 2) > DO_BDCM_ (IC_50_ 9.7 ± 0.0 µg/ml, *n* = 2) > DO_LDCM_ (IC_50_ 16.2 ± 0.0 µg/ml, *n* = 2) > PF_LDCM_ (IC_50_ 132.4 ± 0.0 µg/ml, *n* = 2) > DO_BHEX_ (IC_50_ 185.2 ± 0.0 µg/ml, *n* = 2) (Table 1).

#### Activity of crude extracts against adult* O. ochengi*

**Fig. 3:**
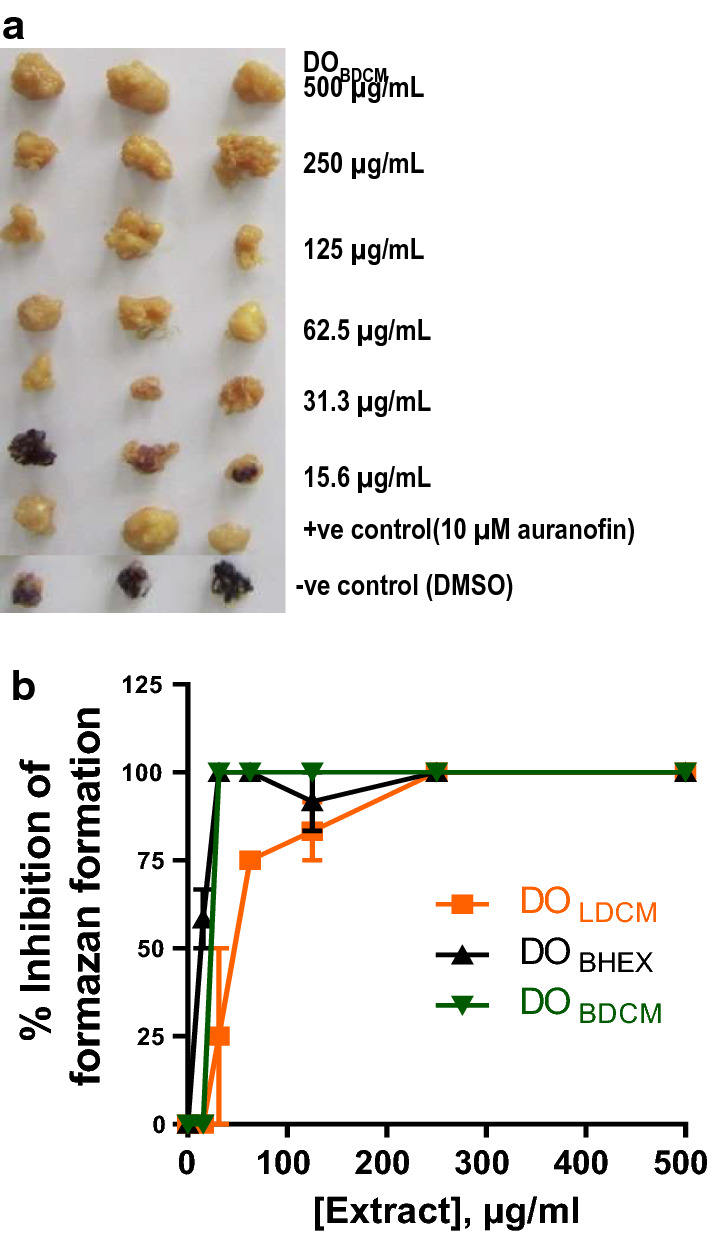
Effects of the three crude extracts of *D. oliveri* identified as active against adult *O. ochengi*. **a** Illustration of the MTT/formazan assay for one of the active extracts, *DO*_*BDCM*_. Each extract was tested in triplicate at concentrations ranging from 15.6 to 500 μg/ml. DO_BDCM_ at concentrations ranging from 31.3 to 500 μg/ml caused a 100% inhibition of purple formazan formation (which correlates with worm death). **b** Concentration-dependent inhibition of formazan formation after 120 h of incubation with *D. oliveri* extracts. Results are plotted as the mean ±  SEM, *n* = 3. The order of potency was: DO_BHEX_ (IC_50_ 13.9 ± 1.7 µg/ml) > DO_BDCM_ (IC_50_ 22.5 ± 0.0 µg/ml) > DO_LDCM_ (IC_50_ 43.3 ± 9.2 µg/ml)

Of the 12 extracts screened, three (DO_BHEX_, DO_LDCM_ and DO_BDCM_) were active against adult *O. ochengi*. Figure 3a illustrates the MTT/formazan assay on DO_BDCM_. All three active extracts showed a concentration-dependent inhibition of formazan formation. The rank order potency was: DO_BHEX_ (IC_50_ 13.9 ± 1.7 µg/ml, *n* = 3) > DO_BDCM_ (IC_50_ 22.5 ± 0.0 µg/ml, *n* = 3) > DO_LDCM_ (IC_50_ 43.3 ± 9.2 µg/ml, *n* = 3). The lowest IC_100_ of 31.3 µg/ml was recorded for extract DO_BHEX_ (Fig. 3b). Extracts DO_BHEX_, DO_BDCM_ and DO_LDCM_ were also active against adult *B. pahangi* and *O. ochengi* mfs; hence, these three extracts were chosen for further investigation.

### Activity of Sep-Pak fractions against adult* B. pahangi*

**Fig. 4:**
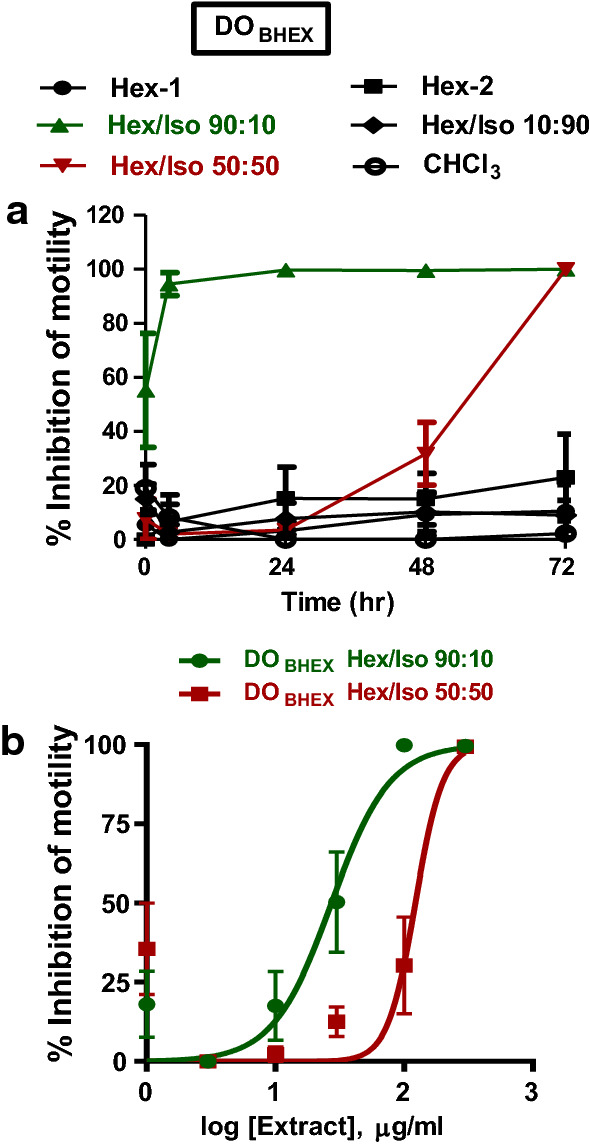
Effects of DO_BHEX_ Sep-Pak fractions on adult *B. pahangi* motility. **a** Time-dependent inhibition of adult *B. pahangi* motility following treatment with DO_BHEX_ fractions from Sep-Pak Silica Plus cartridges. **b** Concentration-dependent inhibition of adult *B. pahangi* motility following treatment with active DO_BHEX_ Sep-Pak fractions. Results are plotted as the mean ± SEM, *n* = 4. Active DO_BHEX_ Sep-Pak fractions are given in green (DO_BHEX_ Hex/Iso 90:10) and red (DO_BHEX_ Hex/Iso 50:50) while inactive fractions are given in black. DO_BHEX_ Hex/Iso 90:10 (IC_50_ 27.3 ± 1.2 µg/ml) was more potent than DO_BHEX_ Hex/Iso 50:50 (IC_50_ 122.2 ± 1.3 µg/ml)

**Fig. 5:**
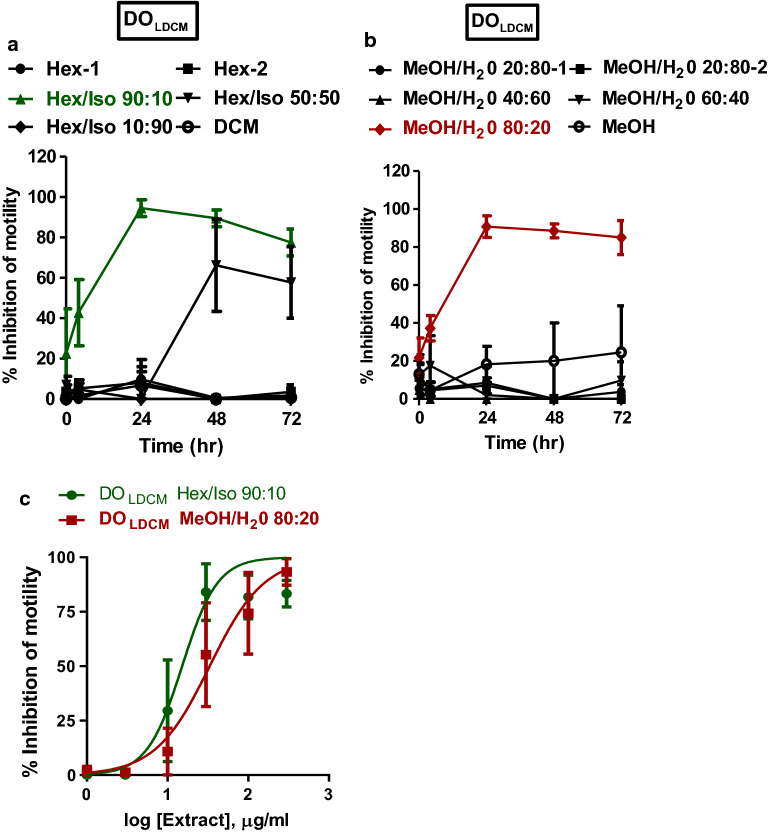
Effects of DO_LDCM_ Sep-Pak fractions on adult *B. pahangi* motility. **a** Time-dependent inhibition of adult *B. pahangi* motility following treatment with DO_LDCM_ fractions from Sep-Pak Silica Plus cartridges. **b** Time-dependent inhibition of adult *B. pahangi* motility following treatment with DO_LDCM_ fractions from Sep-Pak C-18 Plus cartridges. **c** Concentration-dependent inhibition of adult *B. pahangi* motility following treatment with active DO_LDCM_ Sep-Pak fractions. Results are plotted as the mean ± SEM, *n* = 4. Active DO_LDCM_ Sep-Pak fractions are given in green (DO_LDCM_ Hex/Iso 90:10) and red (DO_LDCM_ MeOH/H_2_O 80:20) while inactive fractions are given in black. DO_LDCM_ Hex/Iso 90:10 (IC_50_ 15.2 ± 1.3 µg/ml, *n* = 4) was more potent than DO_LDCM_ MeOH/H_2_O 80:20 (IC_50_ 33.3 ± 1.3 µg/ml, *n* = 4)

Extracts DO_BHEX_, DO_LDCM_ and DO_BDCM_ were subjected to bioassay-guided fractionation by subjecting the extracts to SPE using Sep-Pak Plus cartridges. The resulting fractions were initially tested for activity against adult *B. pahangi* in quadruplicate at a single concentration of 300 µg/ml for 3 days (72 h) (Fig. 4a, 5a, b, 6a, b). Sep-Pak fractions that showed ≥ 75% inhibition of motility at 72 h of incubation were selected for IC_50_ determination; these extracts were DO_BHEX_ eluted with 90% hexane in 10% isopropanol (DO_BHEX_ Hex/Iso 90:10), DO_BHEX_ eluted with 50% hexane in 50% isopropanol (DO_BHEX_ Hex/Iso 50:50), DO_LDCM_ eluted with 90% hexane in 10% isopropanol (DO_LDCM_ Hex/Iso 90:10) and DO_LDCM_ eluted with 80% methanol in 20% water (DO_LDCM_ MeOH/H_2_O 80:20). The rank order of potency for the active Sep-Pak fractions was: DO_LDCM_ Hex/Iso 90:10 (IC_50_ 15.2 ± 1.3 µg/ml, *n* = 4) > DO_BHEX_ Hex/Iso 90:10 (IC_50_ 27.3 ± 1.2 µg/ml, *n* = 4) > DO_LDCM_ MeOH/H_2_O 80:20 (IC_50_ 33.3 ± 1.3 µg/ml, *n* = 4) > DO_BHEX_ Hex/Iso 50:50 (IC_50_ 122.2 ± 1.3 µg/ml, *n* = 4) (Figs. 4b, 5c).

**Fig. 6:**
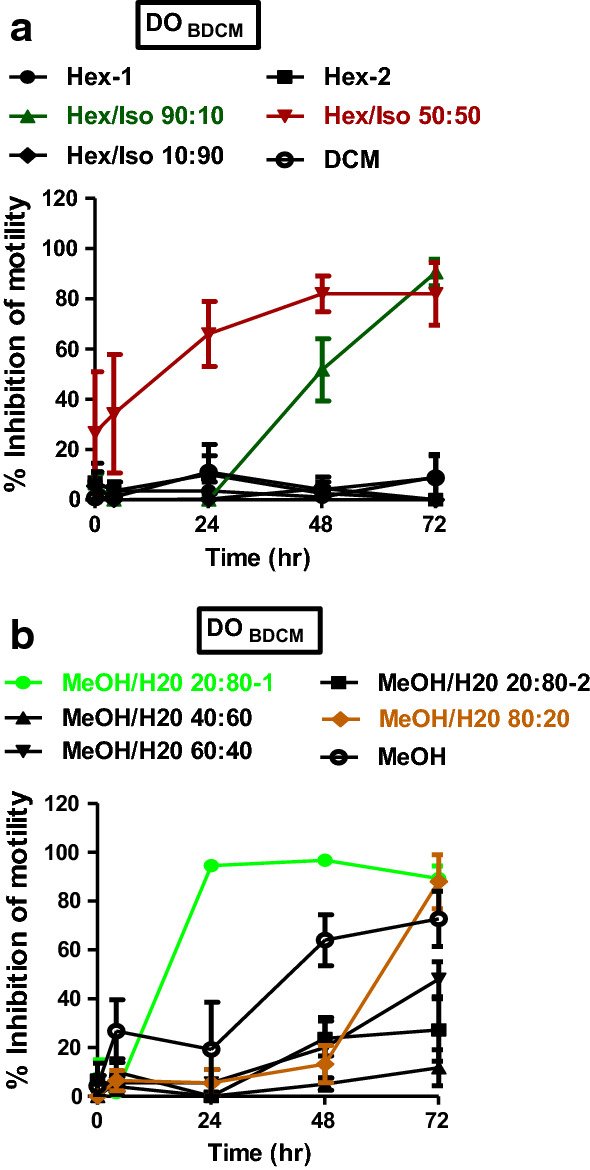
Effects of DO_BDCM_ Sep-Pak fractions on adult *B. pahangi* motility. **a** Time-dependent inhibition of adult *B. pahangi* motility following treatment with DO_BDCM_ fractions from Sep-Pak Silica Plus cartridges. **b** Time-dependent inhibition of adult *B. pahangi* motility following treatment with DO_BDCM_ fractions from Sep-Pak C-18 Plus cartridges. Results are plotted as the mean ±  SEM, *n* = 4. Active DO_BDCM_ Sep-Pak fractions are given in green (DO_BDCM_ Hex/Iso 90:10), red (DO_BDCM_ Hex/Iso 50:50), light green (DO_BDCM_ MeOH/H_2_O 80:20-1) and brown (DO_BDCM_ MeOH/H_2_O 80:20-2) while inactive fractions are given in black

### Activity of HPLC fractions against adult* B. pahangi*

**Fig. 7:**
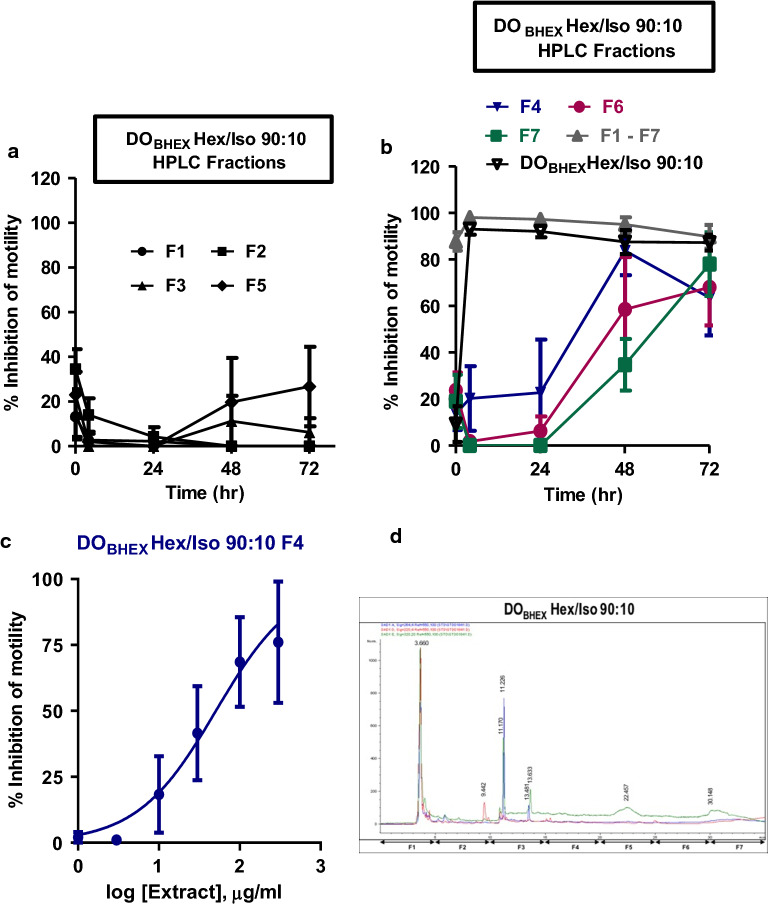
Inhibition of adult *B. pahangi* motility following treatment with DO_BHEX_ HPLC fractions (*F1*–*F7*). **a** Time-dependent effects of inactive DO_BHEX_ HPLC fractions on adult *B. pahangi* motility. **b** Time-dependent inhibition of adult *B. pahangi* motility by active DO_BHEX_ HPLC fractions. **c** Concentration-dependent inhibition of adult *B. pahangi* motility following treatment with the active DO_BHEX_ HPLC fraction, DO_BHEX_ Hex/Iso 90:10 F4. Results are plotted as the mean ± SEM, *n* = 4. DO_BHEX_ Hex/Iso 90:10 F4 IC_50_ = 50.7 ± 1.0 µg/ml. **d** HPLC chromatogram of DO_BHEX_ Hex/Iso 90:10. Fractions were collected every 5 min over a period of 35 min.* HPLC* High-performance liquid chromatography

**Fig. 8:**
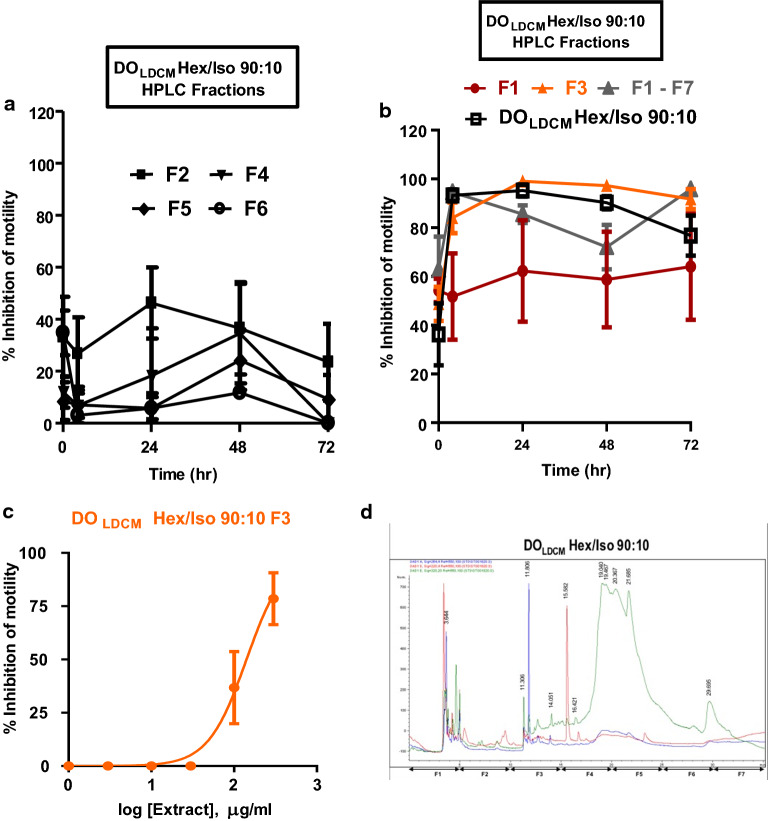
Inhibition of adult *B. pahangi* motility following treatment with DO_LDCM_ HPLC fractions (*F1*–*F7*). **a** Time-dependent effects of inactive DO_LDCM_ HPLC fractions on adult *B. pahangi* motility. **b** Time-dependent inhibition of adult *B. pahangi* motility by active DO_LDCM_ HPLC fractions. **c** Concentration-dependent inhibition of adult *B. pahangi* motility following treatment with the active DO_LDCM_ HPLC fraction, DO_LDCM_ Hex/Iso 90:10 F3. Results are plotted as the mean ± SEM,* n* = 4. DO_LDCM_ Hex/Iso 90:10 F3 IC_50_ = 141.5 ± 1.1 µg/ml. **d** HPLC chromatogram of DO_LDCM_ Hex/Iso 90:10. Fractions were collected every 5 min over a period of 35 min

The most active Sep-Pak fractions from DO_BHEX_ (DO_BHEX_ Hex/Iso 90:10 [IC_50_ 27.3 ± 1.2 µg/ml, *n* = 4]) and DO_LDCM_ (DO_LDCM_ Hex/Iso 90:10 [IC_50_ 15.2 ± 1.3 µg/ml, *n* = 4]) were selected for further fractionation using HPLC. These extracts were chosen because they were the most active against adult *O. ochengi* and adult *B. pahangi*, respectively (Table 1). Seven HPLC subfractions (F1–F7) from each of the two Sep-Pak fractions were generated and again tested for adult *B. pahangi* activity at an initial concentration of 300 µg/ml (Figs. 7a, b, 8a, b) in order to eliminate inactive subfractions and select only the active ones for IC_50_ determination (DO_BHEX_ Hex/Iso 90:10 F4 and DO_LDCM_ Hex/Iso 90:10 F3) (Figs. 7c, 8c). We observed DO_BHEX_ Hex/Iso 90:10 F4 (IC_50_ 50.7 ± 1.0 µg/ml, *n* = 4) to be more potent than DO_LDCM_ Hex/Iso 90:10 F3 (IC_50_ 141.5 ± 1.1 µg/ml, *n* = 4).

### Cytotoxicity of active extracts

Of all the 12 active extracts evaluated at an initial single high concentration of 250 µg/ml for toxicity to N27 cells, extracts DO_BHEX_ and PF_BDCM_ showed no cytotoxicity when compared to the untreated controls. In contrast, all of the other ten extracts tested showed a significant reduction (*P* < 0.001) in cell viability compared to the untreated control. These ten extracts were further tested at eight serially diluted concentrations (500 to 3.91 µg/ml) in order to determine their IC_50_ values. Comparing the IC_50_ values for N27 cells with that for parasites revealed that only extract PF_LDCM_ was more toxic to N27 cells than to parasites (Table 2). All of the other extracts were more selective for parasites than for N27 cells, as revealed by their respective IC_50_ values, with an SI of > 1 (note: the higher the SI value, the more toxic the extract is to parasites than to cells and the ‘safer’ or more potentially useful the extract is for therapeutic drug development). Extracts DO_BHEX_ and PF_LMeOH_, respectively, recorded the highest SI for adult worms (SI > 18.0) and mfs (SI 37.6).

**Table 2:** Selectivity index values of active extracts of *D. oliveri* and *P. febrifugum*

Extracts	IC_50_ N27 cells (µg/ml)	*O. ochengi*				*B. pahangi*	
		Microfilariae		Adult		Adult	
		IC_50_ (µg/ml)	SI^a^	IC_50_ (µg/ml)	SI^a^	IC_50_ (µg/ml)	SI^a^
DO_LHEX_	Tbd	Inactive	/	Inactive	tbd	89.8	/
DO_LDCM_^b^	222.8	16.2	13.8	43.3	5.1	6.1	36.5
DO_LMeOH_	Tbd	Inactive	/	Inactive	/	81.9	/
DO_BHEX_^b^	> 250	185.2	> 1.3	13.9	> 18.0	59.0	> 4.2
DO_BDCM_^b^	224.6	9.7	23.2	22.5	10.0	6.3	35.7
DO_BMeOH_	/	Inactive	/	Inactive	/	44.0	/
PF_LHEX_	/	Inactive	/	Inactive	/	153.7	/
PF_LDCM_	35.1	132.4	0.3	Inactive	/	< 300	/
PF_LMeOH_	331.1	8.8	37.6	Inactive	/	Inactive	/
PF_BHEX_	47.0	4	11.8	Inactive	/	< 300	/
PF_BDCM_	> 250	7.2	> 34.7	Inactive	/	< 300	/
PF_BMeOH_	98.2	6.6	14.9	Inactive	/	< 300	/

^a^Selectivity index (SI) is calculated as IC_50_ N27 cells/IC_50_ parasite

^b^Extracts DO_BHEX_, DO_LDCM_ and DO_BDCM_ were the only plant extracts of the 12 active extracts identified which were active in all three biological assays used in this study (*O. ochengi* microfilariae, adult *O. ocheng*i or adult *B. pahang*). *Tbd* To be determined

### Phytochemical analysis of active extracts

All active crude extracts were screened for the presence of phytochemicals as described in detail in the Methods section. The presence (+) and absence (−) of the different phytochemicals screened are shown in Table 3. Steroids and saponins were found to be present in all three active extracts (DO_BHEX_, DO_LDCM_ and DO_BDCM_), while cuomarins and tannins were absent. In addition, DO_LDCM_ and DO_BDCM_ also contained flavonoids and cardiac glycosides while DO_BHEX_ contained alkaloids.

**Table 3:** Phytochemical analysis of active extracts of *D. oliveri* and *P. febrifugum*

Class of compound	*D. oliveri* extracts			*P. febrifugum* extracts				
	DO_LDCM_	DO_BHEX_	DO_BDCM_	PF_LDCM_	PF_LMeOH_	PF_BHEX_	PF_BDCM_	PF_BMeOH_
Alkaloids	−	+	−	+	+	+	+	+
Steroids	+	+	+	+	+	+	+	+
Flavonoids	+	−	+	+	+	+	+	+
Saponins	+	+	+	+	+	+	+	+
Tannins	−	−	−	+	+	−	−	+
Cuomarins	−	−	−	−	−	−	−	−
Cardiac glycosides	+	−	+	−	+	+	−	−

Key: (+) = present, (−) = absent

## Discussion

Onchocerciasis and LF are two major NTDs whose control is hampered by the lack of suitable adulticides. There is a pressing need for new compounds for drug development against the parasites that cause these diseases. In Cameroon, there is an abundance of medicinal plants whose bioactivity has yet to be explored. In the present study, *D. oliveri* and *P. febrifugum* were collected from Bambui, North West Region of Cameroon based on ethnopharmacological information provided by traditional healers. Only a few scientific studies on the pharmacological activities of *D. oliveri* and *P. febrifugum* extracts have been reported. The methanolic extracts of *D. oliveri* isolated from the stem bark and leaves possess neuromuscular blocking actions on rat skeletal muscle [39]. The* n*-butanol soluble fraction of the acetone methanolic extract of *D. oliveri* bark acts as a non-competitive antagonist for muscarinic receptors on isolated rat bladder smooth muscle [40]. The *n*-butanol soluble fraction and fractions of the aqueous ethanolic extract of *D. oliveri* leaves show activity against *Staphylococcus aureus* and the fungus *Tricophyton rubrum* and cause a dose-dependent relaxation of isolated rabbit jejunum as well as inhibition of castor oil-induced diarrhea in mice [41, 42]. Extracts isolated from *P. febrifugum* leaves and roots show broad-spectrum antimicrobial activity against bacteria and fungi [43, 44]. *Psorospermum febrifugum* extracts also show significant antineoplastic activity against tumor cells* in vitro* and in mice. In earlier studies, bioassay-guided fractionation of these extracts identified a series of xanthones as being responsible for this antineoplastic activity [45–50].

Here, we report for the first time, empirical evidence of the anthelmintic activity of *D. oliveri* and *P. febrifugum* as alternatives for the treatment of onchocerciasis and LF. All 12 crude extracts screened showed time- and concentration-dependent activity in one or more of the three bioassays (*O. ochengi* mfs, adult *O. ocheng*i or adult *B. pahangi*). Only three (DO_BHEX_, DO_LDCM_ and DO_BDCM_) of these 12 extracts were active in all three biological assays used in this study, implying these extracts have potential as sources of lead compounds for broad-spectrum filaricidal activity. This observation, in addition to the low adult worm IC_50_ values (6.1–59.0 µg/ml) recorded by DO_BHEX_, DO_LDCM_ and DO_BDCM_ and their lack of cytotoxicity encouraged us to select these extracts for further investigation (Tables 1, 2). IC_50_ values varied with the different stages of the two nematodes, suggesting that these extracts may have different targets in the parasites. The highest SI was recorded with the DO_BDCM_ extract. The observed low cytotoxicity coupled to the efficacy of these extracts could account for their continuous use in the local management of these helminth infections over the years, raising the hope of the likelihood of success rate if these products are further developed as a cure for filariasis.

Initial fractionation of DO_BHEX_, DO_LDCM_ and DO_BDCM_ was achieved using Sep-Pak Plus cartridges, and the most promising fractions were chosen for further investigation using semi-preparative HPLC. For each active Sep-Pak fraction, seven HPLC fractions were collected and tested for activity against *B. pahangi* and the IC_50_ determined from concentration–response plots. We noted that the potency of the extracts varied during the course of the fractionation process and that the activity was similar to that of the parent Sep-Pak fraction when all of the HPLC fractions were combined. This led to the conclusion that the reduction in activity in the HPLC fractions may be attributed to the presence of multiple bioactive compounds acting together and that these compounds are contained in two or more HPLC fractions. In addition to this synergistic effect, the extracts might be unstable, consequently unable to withstand the fractionation process.

The phytochemicals present in our promising extracts (Table 3) suggest that the bioactive compounds in the extracts may include alkaloids, steroids, flavonoids, saponins or cardiac glycosides. The presence of one or more of these phytochemicals has been reported in the following plants with demonstrated activity against *O. ochengi*: *Margaritaria discoidea*, *Homalium africanum*, *Craterispermum laurinum*, *Morinda lucida*, *Tragia benthami*, *Piper umbellatum* and *Annona senegalensis* [32, 33, 51]. Also, published data on the anthelmintic activity for some phytochemicals, such as alkaloids, flavonoids and saponins, are available [52–54].

## Conclusions

Our results justify the ethnomedicinal use of *D. oliveri* and *P. febrifugum* and reaffirm that medicinal plant extracts are important sources of compounds with potent filaricidal activity. The further fractionation of these extracts would allow for the isolation and identification of lead compounds for therapeutic drug discovery and development.

## Supplementary Information

**Additional file 1: Table S1.** Classification of plants screened. **Table S2.** Percentage (%) yield of plant extracts.

## Data Availability

All data generated or analysed during this study are included in this published article [and its supplementary information files].
