# Supplementary material for: Filaricidal activity of Daniellia oliveri and Psorospermum febrifugum extracts
Source: Parasit Vectors. 2021 Jun 7;14:305. doi: 10.1186/s13071-021-04759-6 (PMC8186089; doi:10.1186/s13071-021-04759-6)
Supplement: Supplementary file 1 — Additional file 1: Table S1. Classification of plants screened. Table S2. Percentage (%) yield of plant extracts. [file 13071_2021_4759_MOESM1_ESM.docx]

**Table S1. Classification of plants screened**

| **Family** | **Scientific name** | **Common name** | **Traditional uses** | **Plant parts used** | **Code adopted** |
| --- | --- | --- | --- | --- | --- |
| Caesalpiniaceae | *Daniellia oliveri* | Kahi | Treatment of all worm infections (including river blindness, lymphatic filariasis) and stomach upset | Leaves (L) and stem bark (B) | DO |
| Hypericaceae | *Psorospermum febrifugum* | Sawayki | Treatment of onchocerciasis and rashes of any kind | Leaves (L) and stem bark (B) | PF |

**Table S2. Percentage (%) yield of plant extracts**

| **Plant** | **Dry weight (Wt) of plant part (g)** | **Hexane**  **(HEX)** | | **Dichloromethane (DCM)** | | **Methanol**  **(MeOH)** | |
| --- | --- | --- | --- | --- | --- | --- | --- |
|  |  | **Wt (g)** | **% yield** | **Wt (g)** | **% yield** | **Wt (g)** | **% yield** |
| *Daniella oliveri* | 750.0 (leaves) | 17 | 2.27 | 12 | 1.6 | 16.8 | 2.24 |
|  | 500.0  (stem bark) | 4.6 | 0.92 | 4.3 | 0.86 | 52.3 | 10.46 |
| *Psorospermum febrifugum* | 600.0 (leaves) | 3.0 | 0.5 | 10.9 | 1.82 | 51.4 | 8.57 |
|  | 300.0  (stem bark) | 21.7 | 7.23 | 48.1 | 16.03 | 120.2 | 40.07 |
